# Effects of dietary supplementation with alkaline mineral complex on *in vitro* ruminal fermentation and bacterial composition

**DOI:** 10.3389/fvets.2024.1357738

**Published:** 2024-05-22

**Authors:** Siyuan Liu, Biao Xie, Hongjin Ji, Shengli Li

**Affiliations:** State Key Laboratory of Animal Nutrition, Beijing Engineering Technology Research Centre of Raw Milk Quality and Safety Control, College of Animal Science and Technology, China Agricultural University, Beijing, China

**Keywords:** lactating ruminants, rumen preference parameters, additive concentration, subacute rumen acidosis, dairy cows

## Abstract

**Introduction:**

Dairy industry growth faces challenges in China due to inadequate forage, leading to high-concentrate diets and potential rumen issues. Buffering agents, like sodium bicarbonate, play a crucial role in stabilizing rumen pH. Alkaline Mineral Complex (AMC), a liquid additive with a pH of 14, shows promise in supporting dairy cow health and mitigating heat stress through ionization.

**Methods:**

This experiment was aimed to study the effect of adding AMC to total mixed ration (TMR) on *in vitro* ruminal fermentation and bacterial composition. AMCat 1, 2, 4, and 8 mL/kg was added to the substrate (0.5 g TMR). Nutrient digestibility was measured after 48 h fermentation, and fermentation parameters and microbial composition were measured after 48 h fermentation.

**Results and discussion:**

The results of the experiment indicated that: The different concentrations of AMC showed a significant impact on time taken for gas production to reach 1/2 of the total gas production (HT) parameters (*p* < 0.05). Linear pH increase occurs at 6 and 24 h with rising AMC concentration (*p* < 0.05), showing a quadratic trend at 12 h (*p* < 0.05). The optimal buffering effect on rumen acid-base balance was observed at a 2 mL/kg concentration of AMC. Microbial diversity analysis indicated that there was no significant change in α-diversity with different AMC concentrations (*p* > 0.05). The microbial level demonstrated no significant difference in species diversity of rumen fluid bacteria among the various AMC concentration treatment groups compared to the control group, further supporting that the advantage of adding AMC in stabilizing the rumen environment without altering the structure of the rumen microbiota. Besides, the addition of AMC significantly increased the concentrations of acetate, propionate, total fatty acids (TVFA), and NH3-N, suggesting that AMC contributed to enhancing the energy and nitrogen utilization efficiency in ruminants. Based on the above detection indicators, we recommend that the most favorable concentration is 2 mL/kg.

## 1 Introduction

The expansion of the dairy industry has been aided by the increasing demand for dairy products. However, the lack of high-quality forage in China makes it difficult to measure the nutritional requirement of lactating ruminants. Many ranches have decided to increase the proportion of concentrate feed to meet this requirement. High-concentrate diets have a relatively low effective fiber content and can easily induce subacute rumen acidosis (SARA) in ruminants (1). SARA is a metabolic disorder in animals, and it is characterized by rumen fluid pH values that are consistently < 5.8 and persist for more than 4 h after feed consumption (2, 3). This phenomenon, which the rumen is unable to effectively neutralize, is mainly caused by an excessive intake of highly fermentable carbohydrates. It severely impairs the lactation performance of dairy cows, leading to substantial financial losses for the pasture. Additionally, it can also cause other diseases such as mastitis, which endangers animal health. Hence, it is critical to address the adverse effects of high-concentrate diets and emphasize the importance of balancing the physiological wellbeing and productivity of lactating dairy cows.

A buffer is a type of compound or mixture that enhances the acid-base buffering capacity of a solution. In ruminant animal production, it is important to maintain the pH in the rumen at a stable level of 5.8–6.2 to support the activity of rumen microorganisms (4). To maintain the normal rumen fermentation performance of cow-fed high-concentrate diets, strongly alkaline and weakly acidic salts are typically used as buffering agents to prevent rumen acidosis and improve their productivity. Some buffering agents, commonly used in ruminants both domestically and internationally, include sodium bicarbonate, magnesium oxide, sodium acetate, sodium butyrate, calcium carbonate, and other minerals. Composite buffering agents have more efficient pH regulation ability than single buffering agents. Neiderfer et al. revealed that the supplementation of the daily diet of lactating cows with CaCO_3_, MgO, and coated NaHCO_3_ effectively maintained their rumen fluid pH (5). Similarly, Snyder et al. observed that the addition of NaHCO_3_ and its composite buffering agent to the diet of lactating cows enhanced their milk production and milk fat percentage (6).

Alkaline mineral complex (AMC) is a colorless, tasteless, and non-toxic complex alkaline ion mixture with a pH of 14 (7–9). It is a liquid feed additive that helps cows maintain the acid-base balance of ruminal fluids, preserves the normal function of cellular ion pumps, and improves immunity. It activates immune cells by enhancing neuromuscular physiological information transmission and physiological regulatory functions, thereby alleviating heat stress in dairy cows. The ions generated by the ionization of AMC solution jointly regulate H^+^ in the rumen. Despite its limited application in dairy cows, this composite buffering agent has a promising potential (9).

Therefore, the objective of this study was to investigate the effects of different concentrations of AMC on fermentation characteristics and bacterial composition *in vitro* to establish the optimal additive concentration for large-scale feeding applications in dairy herds.

## 2 Materials and methods

### 2.1 Animals and their feeding management

The rumen fluid was collected from three healthy, mid-lactating, and rumen-cannulated Holstein dairy cows with similar milk yield (26 ± 1.63 kg/d) from Zhongdi Dairy Holdings Co., Ltd. (Beijing, China). The dairy cows had *ad libitum* access to feed and water. The total mixed ratio (TMR) was fed to the cows three times daily (07:00, 14:00, and 19:00), and the cows were milked three times a day at 06:30, 13:30, and 18:30. All the animal procedures were approved by the Institutional Animal Care and Use Committee of China Agricultural University (approval number: AW61902202-1-4).

### 2.2 Experimental design

#### 2.2.1 Fermentation substrates

All fermentation substrates (donor cows' TMR) were crushed and kept in the oven at 65°C for 48 h (10, 11). After drying, the samples were crushed and sieved through a 1 mm screen for subsequent fermentation processes, and the chemical composition was determined using the Association of Official Analytical Chemists (AOAC) methods (12). The ingredients and nutrient compositions of all the fermentation substrates are shown in Table 1.

**Table 1:** Fermentation substrate composition and nutrients (dry matter basis, %).

**Items^a^**	**Contents**
Ingredients, % of DM	
Alfalfa hay	3.74
Alfalfa silage	1.72
Whole corn silage	32.31
Steam-flaked corn	14.86
Corn	10.18
Whole cottonseed	1.77
Extruded soybean meal	12.58
Soybean hull	10.72
DDGS	3.77
Fat powder	0.75
Corn gluten meal	2.56
Molasses	0.32
NaHCO_3_	0.57
Premix	4.14
Total	100
Nutrient levels,% of DM	
NE_L_ (MJ/kg)^b^	7.28
Concentrate to forage ratio	47:53
CP	16.45
Ether extract	5.1
Ash	6.7
NDF	40.94
ADF	30.81

^a^EE, ether extract; CP, crude protein; NDF, neutral detergent fiber; ADF, acid detergent fiber.

^b^NE_L_, net energy of lactation, these data in the diet are calculated by multiplying the net energy produced by each raw material and its proportion in the diet.

#### 2.2.2 AMC

The AMC used in this study was provided by Beijing Jinaer Biotechnology Co., Ltd. The AMC utilizes zinc oxide and germanium compounds as cell activators in combination with sodium and potassium compounds. The elements, such as Si, Ge, K, and Zn, in the alkaline solution remain in ionic and water-soluble states, thereby maintaining a weak alkaline internal environment for the animals. The composition and mineral ion contents are shown in Tables 2, 3, respectively.

**Table 2:** The composition of alkaline mineral complex (AMC) water concentrate.

**Ingredients**	**Chemical formula**	**Contents (mg/L)**
Sodium metasilicate pentahydrate	5H_2_O·Na_2_SiO_3_	200
Potassium bicarbonate	KHCO3	100
Zinc oxide	ZnO	0.01
Bis-(carboxyethylgermanium) sesquioxide	Ge-132	0.001

**Table 3:** The mineral ion content of AMC water.

**Ions**	**Calculated contents (mg/L)**
SiO3^2−^	179.25
Na^+^	108.49
K^+^	97.50
Zn^2+^	0.02
Ge^4+^	0.0005
HCO${\;}_{3}^{-}$	152.50

#### 2.2.3 Rumen fluid collection

The rumen fluid was collected 2 h after morning feeding. The collected rumen fluid was filtered through four layers of gauze and placed in a thermos for quick return to the laboratory. It was then transferred into a 4 L beaker filled in advance with CO_2_ at 39°C in a water bath (11).

#### 2.2.4 In vitro degradability

The test was divided into five groups with three replicates for measuring gas production and pH. The substrate degradation experiment was performed using five replicates per group. Except for the control group, AMC was added to each group at 1, 2, 4, and 8 mL/kg of the substrate.

For every *in vitro* gas production experiment, a total of 500 mg fermentation substrate, 25 mL of rumen fluid, and 50 mL of buffer (13) were added to a 120 mL anaerobic fermentation bottle. For the other fermentation bottles, 3 g samples from each treatment were individually placed into 250 mL glass bottles, which contained 150 mL of buffer solution and 75 mL of rumen fluid.

Each bottle was immediately sealed with butyl rubber stoppers and Hungate's screw caps after the addition of the experimental samples, and nitrogen was injected until oxygen was discharged.

The gas production bottles were placed inside a 39°C constant temperature incubator and were immediately connected to the corresponding gas channels of the AGRS-III system according to the pre-arranged inoculation order (14). Furthermore, the gas production (GP) was automatically recorded throughout the 48 h of fermentation. All the bottles were kept in a thermostatic incubator to ferment continuously for 6, 12, 24, 36, and 48 h. After 24 and 48 h of fermentation, the bottles were removed from the incubator. At every time point, the fermentation was halted by placing the bottles in a mixture of ice and water for 15 min.

### 2.3 Sample collection and measurement

The pH was measured five times at the five fermentation time points. After incubation, the contents of each bottle were filtered using a filter with 42 μm pores (sized 80 × 150 mm). As described in previous studies (12, 13), the volatile fatty acid concentration in the supernatant was determined using gas chromatography, and NH_3_-N was measured using a spectrophotometer. The remaining samples were kept at −80°C. One sample was used for further microbial community analysis, while others were used to measure microbial crude protein (MCP).

### 2.4 DNA extraction and determination

Total microbial genomic DNA was extracted using a kit from MP Biomedicals, Solon, OH, USA, and the NanoDrop^®^ ND-2000 spectrophotometer (Thermo Scientific Co., Ltd., Waltham, Massachusetts, USA) was used to assess the DNA purity and concentration. Additionally, DNA integrity was assessed using 1% agarose gel electrophoresis.

The V3-V4 region of the *16S rRNA* gene was amplified with forward primer 338F (5′-ACTCCTACGGGAGGCAGCAG-3′) and 806R (5′-GGACTACH VGGGTWTCTAAT-3′) through the polymerase chain reaction (PCR). For each sample, three PCR replicates were mixed, and 5 mL of the PCR product from each sample was detected using 2% agarose gel electrophoresis (15, 16). The PCR products were purified using an AxyPrep DNA Gel Extraction Kit (AP-GX-250, Axygen Biosciences, Union City, USA) and were quantified using a quantum fluorometer (E6150, Promega, WI, USA).

Finally, the amplicons were sequenced using a MiSeq pe300 platform (Illumina, Inc., San Diego, California, USA). Quality control (QC) and splicing of the original sequence and ASV representative sequences were clustered according to 97% similarity using UPARSE software (version7.0.1090,http://drive5.com/uparse/), and UCHIME software (version7.0, http://www.drive5.com/usearch/) was applied to eliminate the chimera (17).

The sequences containing more than 10% unknown nucleotides were excluded from the subsequent analysis. The paired-end clean tags were combined into raw tags using FLASH v. 1.2.11 software, following the methodology outlined by Magoč and Salzberg (18). The merging process had a minimum overlap of 10 bp, and a mismatch rate of 0.1 was used to generate Fasta sequences.

The sequencing data were saved in the form of a FASTQ file. The sequences were subjected to ASV clustering at a 97% similarity threshold using UPARSE 7.1 (19), and the chimeras were removed. The taxonomy annotations of ASV of species were classified and annotated using the Ribosomal Database Project (RDP) (http://rdp.cme.msu.edu/) (17) against the Silva 16S rRNA gene database (v138) with a confidence threshold of 70%.

### 2.5 Calculation and analyses

The corresponding cumulative gas production (GP, mL/g, dry matter basis) was fitted non-linearly with each fermentation time using the exponential model described by France et al. (20) as follows:


(1) $$\begin{array}{l} GP_{t}=A[1-e^{-c\left(t-lag\right)} \end{array}$$

GP_t_: where GP_t_ (mL) is the total gas production (mL/g dietary DM) over time t, A is the maximum gas production of the fermentation substrate at a gas production rate c (h^−1^) (mL), and lag is the delay time of fermentation gas production (h).

For the AGPR, the average gas production rate is as follows:


(2) $$\begin{array}{l} AGPR=A\times \frac{c}{log2+c\times lag} \end{array}$$

where A, c, and lag are the same as those in Equation (1).

AGPR, Average gas production rate when half of the ideal maximum gas production is achieved (mL/h).

The test data obtained were preliminarily collated using Excel 2020 and analyzed using the mixed model in SAS 9.4 (21). The standard error (SEM) of the least-squares mean of each measurement indicator was determined using LSMENAS statements, and multiple comparisons were performed using Duncan's test. The minimum significant difference method was used for comparisons when the difference was significant (*p* < 0.05), and 0.05 < *p* < 0.1 indicates that the data have a significant downward or upward trend.

The alpha diversity analysis at the ASV level was conducted using Mothur v1.30.1 (22) software. Differences in the α diversity index between different types were obtained using the Wilcoxon rank sum test.

In the beta diversity analysis, principal component analysis (PCA) based on the Bray-Curtis distance algorithm was used to test for discrepancies in microbial communities at the ASV level between different groups (23). The non-parametric Kruskal–Wallis rank sum test was used to detect the genera with significant differences in abundance between different groups, and the consistency of the differences in different genera was subjected to the Tukey–Kramer test in different subgroups between the groups. Additionally, hypothesis testing was performed to evaluate the genus abundance between multiple groups. These analyses revealed genus information that showed significant differences among the treatment groups.

The data were analyzed using the online platform Majorbio Cloud Platform (www.majorbio.com).

## 3 Results

### 3.1 Gas production kinetics parameters

Table 4 presents an overview of the different concentrations of AMC *in vitro* gas production kinetics parameters. Through data analysis, it was found that HT had a linear trend of growth at different concentrations of AMC (*p* < 0.01), whereas there was no significant difference between the AMC_1_ and AMC_2_ groups and the control group. This indicates that the addition of AMC at a concentration of 1 and 2 mL/kg did not affect the rumen fermentation efficiency.

**Table 4:** Effects of different concentrations of AMC on gas production kinetics parameters.

**Items**	**Groups**					**SEM**	***P*** **-value**		

	**CON**	**AMC**_1_	**AMC**_2_	**AMC**_3_	**AMC**_4_		**G**	**L**	**Q**
GP_48_ (mL)	106.99	123.15	113.04	111.08	106.47	2.246	0.113	0.379	0.067
A (mL)	102.21	117.37	111.69	110.46	103.97	2.337	0.242	0.833	0.052
HT (h)	2.25^c^	2.23^c^	2.45^bc^	2.61^ab^	2.85^a^	0.06	0.001	< 0.001	0.238
AGPR (mL/h)	33.00	39.22	34.28	30.48	23.73	2.134	0.228	0.074	0.162

CON, no supplementation; AMC_1_, the AMC concentration in the substrate is 1 mL/kg; AMC_2_, the AMC concentration in the substrate is 2 mL/kg; AMC_3_, the AMC concentration in the substrate is 4 mL/kg; AMC_4_, the AMC concentration in the substrate is 8 mL/kg; G, group effect; L, linear effect; Q, quadratic effect; GP_48_, the total gas production (mL/g dietary DM) over 48 h; A, the maximum gas production of the fermentation substrate at the gas production rate c (h^−1^); HT, time taken for gas production to reach 1/2 of the total gas production; AGPR, average gas production rate when half of the ideal maximum gas production produced. In peer data, different lowercase letters on the shoulder indicate significant differences (*P* > 0.05), while the same or no letters indicate insignificant differences (*P* > 0.05).

### 3.2 Fermentation parameters

As shown in Table 5, with an increase in AMC concentrations, the pH of the rumen fluid increased linearly at 6 h and 24 h (*p* < 0.05), whereas the pH at 12 h showed a quadratic trend (*p* < 0.05). In addition, different concentrations of AMC had no significant effect on pH at 36 h and 48 h (*p* > 0.05). The data showed that AMC had a good buffering effect before 24 h, and this indicated a stabilizing effect on rumen pH. After 24 h, the rumen pH of both the control and treatment groups tended to stabilize.

**Table 5:** Effects of different concentrations of AMC on the pH *in vitro* fermentation.

**Items**	**Groups**					**SEM**	***P*** **-value**		

	**CON**	**AMC**_1_	**AMC**_2_	**AMC**_3_	**AMC**_4_		**G**	**L**	**Q**
6 h	6.58^c^	6.61^bc^	6.65^ab^	6.61^bc^	6.68^a^	0.012	0.024	0.005	0.964
12 h	6.52^ab^	6.53^a^	6.53^a^	6.50^b^	6.48^c^	0.005	0.001	0.004	0.001
24 h	6.58^b^	6.67^a^	6.65^a^	6.61^ab^	6.67^a^	0.010	0.004	0.047	0.089
36 h	6.49	6.50	6.54	6.51	6.52	0.009	0.500	0.269	0.433
48 h	6.65	6.63	6.67	6.64	6.67	0.011	0.671	0.518	0.585

CON, no supplementation; AMC_1_, the AMC concentration in the substrate is 1 mL/kg;AMC_2_, the AMC concentration in the substrate is 2 mL/kg; AMC_3_, the AMC concentration in the substrate is 4 mL/kg; AMC_4_, the AMC concentration in the substrate is 8 mL/kg; G, group effect; L, linear effect; Q, quadratic effect. In peer data, different lowercase letters on the shoulder indicate significant differences (*P* > 0.05), while the same or no letters indicate insignificant differences (*P* > 0.05).

**Figure 1:**
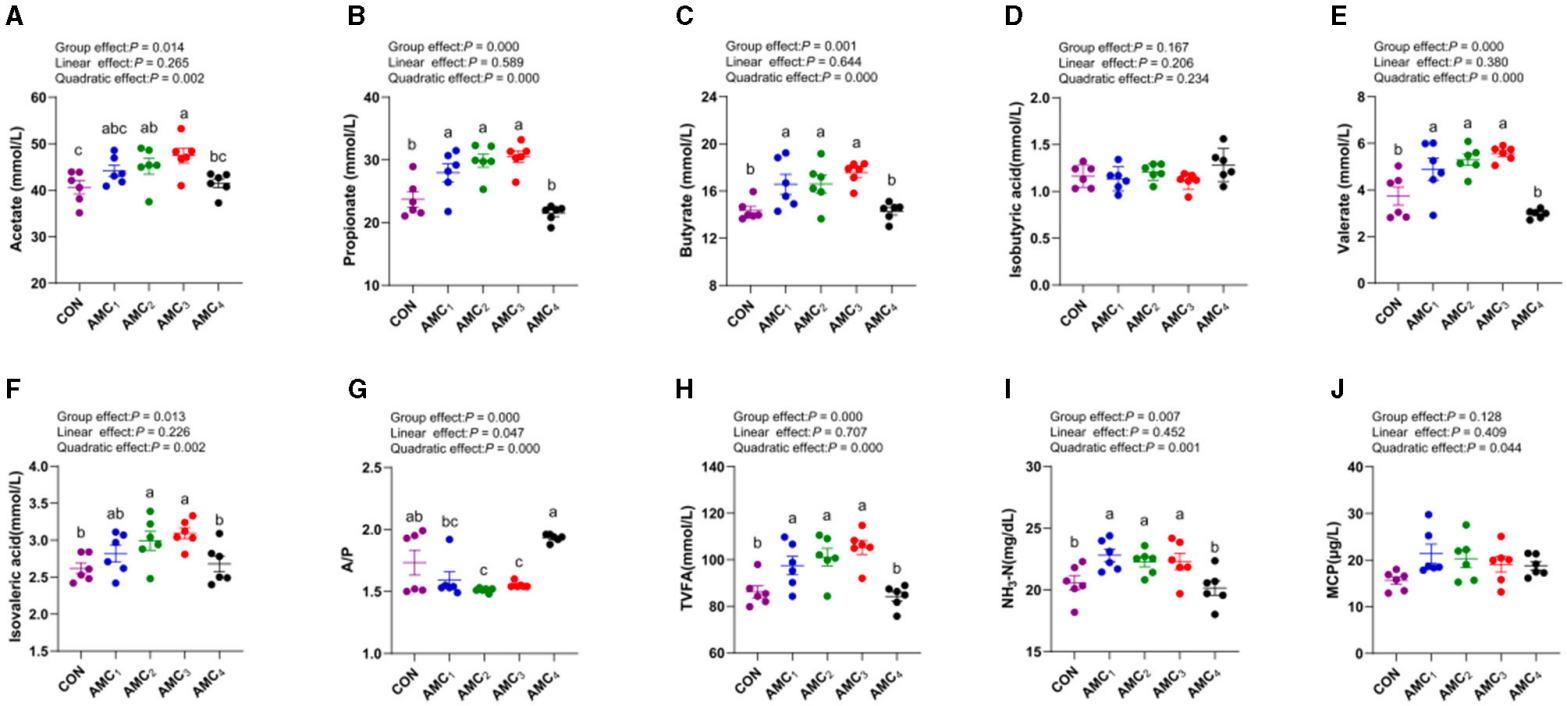
Effects of different concentrations of AMC on fermentation parameters. **(A)** Acetate content; **(B)** Propionate content; **(C)** Butyrate content; **(D)** Isbutyric content; **(E)** Valerate content; **(F)** Isovaleric acid content; **(G)** Acetate/Propionate; **(H)** TVFA-total volatile fatty acids; **(I)** NH_3_-N:Ammonia nitrogen; **(J)** MCP:Microbial protein. CON, no supplementation; AMC_1_, the AMC concentration in the substrate is 1 mL/kg; AMC_2_, the AMC concentration in the substrate is 2 mL/kg; AMC_3_, the AMC concentration in the substrate is 4 mL/kg; AMC_4_, the AMC concentration in the substrate is 8 mL/kg; G, group effect; L, linear effect; Q, quadratic effect; A/P, the ratio of Acetate and Propionate; TVFA, total volatile acids; NH_3_,-N, ammoniacal nitrogen; MCP, microbial crude protein.

Figure 1 shows the impact of the AMC concentration on the profiles of the fermentation parameters during the 48 h of fermentation. The data revealed a quadratic trend for all VFAs and TVFA at different concentrations of AMC, except for isobutyric acid (*p* < 0.05). With an increase in the concentration of AMC, the levels of acetate, propionate, butyrate, valerate, and isovaleric acid increased and subsequently decreased, with the highest value observed in AMC_3_. The ratio of acetate to propionate showed an initial decrease, followed by an increase with increasing concentrations of AMC. The AMC_2_ and AMC_3_ groups tended to exhibit more propionic acid-type fermentation (*p* < 0.05). The influence of different AMC concentrations on MCP was not significant (*p* > 0.05).

### 3.3 *In vitro* degradability

Table 6 presents the effects of different AMC concentrations on nutrient degradability. The results showed that AMC has a negative effect in promoting ADF degradation during *in vitro* fermentation but has no significant effect on DM, NDF, or CP parameters. The acid detergent fiber (ADF) linearly increased with the concentration of AMC (*p* < 0.05).

**Table 6:** Effects of different concentrations of AMC on the nutrient degradability *in vitro* fermentation.

**Items**	**Groups**					**SEM**	***P*** **-value**		

	**CON**	**AMC**_1_	**AMC**_2_	**AMC**_3_	**AMC**_4_		**G**	**L**	**Q**
DM	66.53	68.72	67.98	67.19	66.52	0.391	0.281	0.572	0.083
NDF	54.39	52.32	51.43	52.44	49.91	0.663	0.314	0.066	0.856
ADF	22.30^a^	24.71^b^	25.27^b^	26.75^c^	26.86^c^	0.444	0.002	< 0.001	0.215
CP	62.52	66.70	64.98	66.60	63.45	0.630	0.109	0.648	0.028

DM, dry matter; NDF, neutral detergent fiber; ADF, acid detergent fiber; CP, crude protein; CON, no supplementation; AMC_1_, the AMC concentration in the substrate is 1 mL/kg; AMC_2_, the AMC concentration in the substrate is 2 mL/kg; AMC_3_, the AMC concentration in the substrate is 4 mL/kg; AMC_4_, the AMC concentration in the substrate is 8 mL/kg; G, group effect; L, linear effect; Q, quadratic effect. In peer data, different lowercase letters on the shoulder indicate significant differences (*P* > 0.05), while the same or no letters indicate insignificant differences (*P* > 0.05).

### 3.4 Microbial diversity

**Figure 2:**
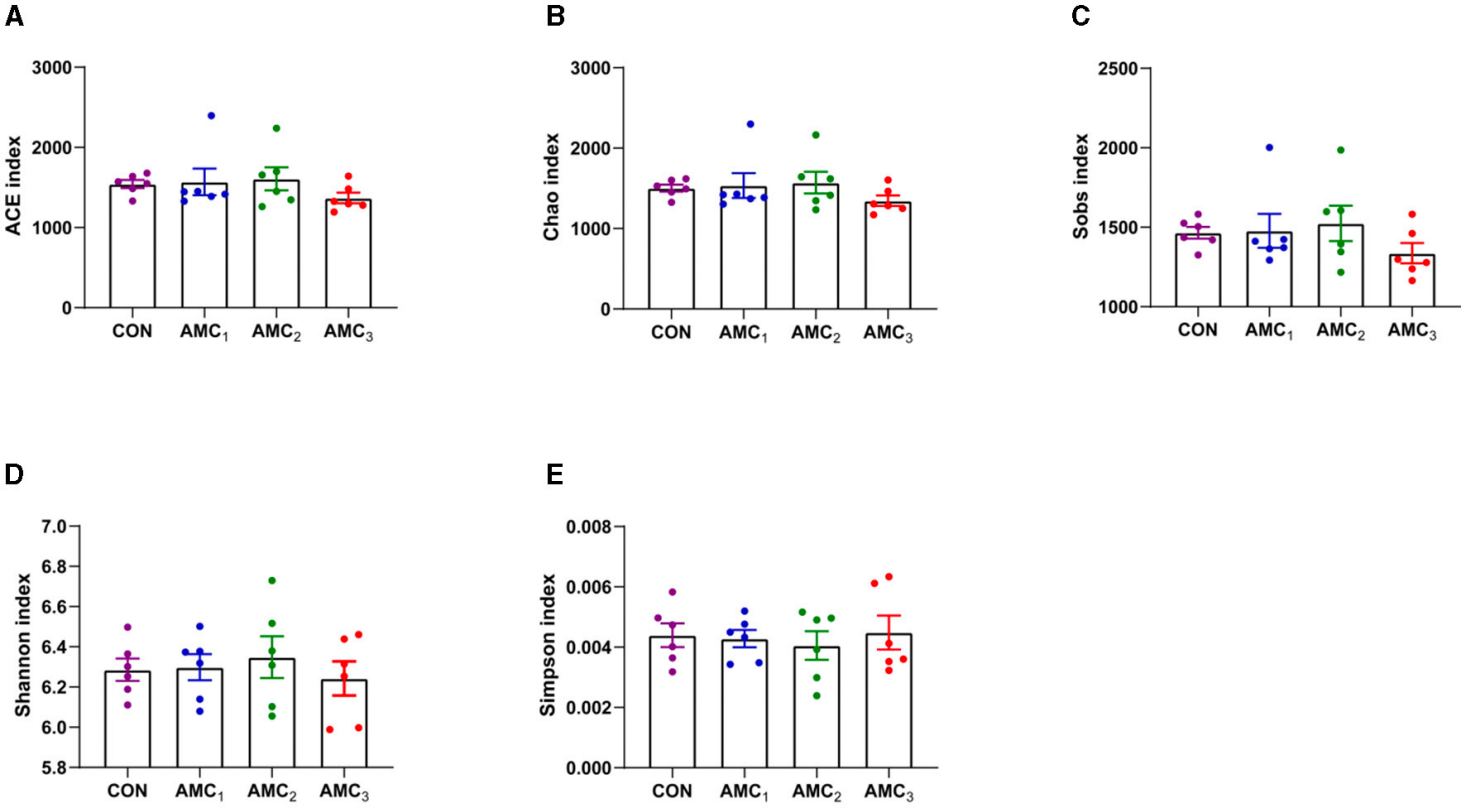
Effects of different concentrations of AMC on the nutrient degradability *in vitro* fermentation. **(A)** ACE diversity index; **(B)** Chao 1 diversity; **(C)** Sobs index; **(D)** Shannon diversity index; **(E)** Simpson index. CON, no supplementation; AMC_1_, the AMC concentration in the substrate is 1 mL/kg; AMC_2_, the AMC concentration in the substrate is 2 mL/kg; AMC_3_, the AMC concentration in the substrate is 4 mL/kg.

Figure 2 shows the effects of different concentrations of AMC on the alpha diversity index *in vitro* fermentation. For the alpha diversity index, there was no significant difference between the treatment group and the CON group (*p* > 0.05).

**Figure 3:**
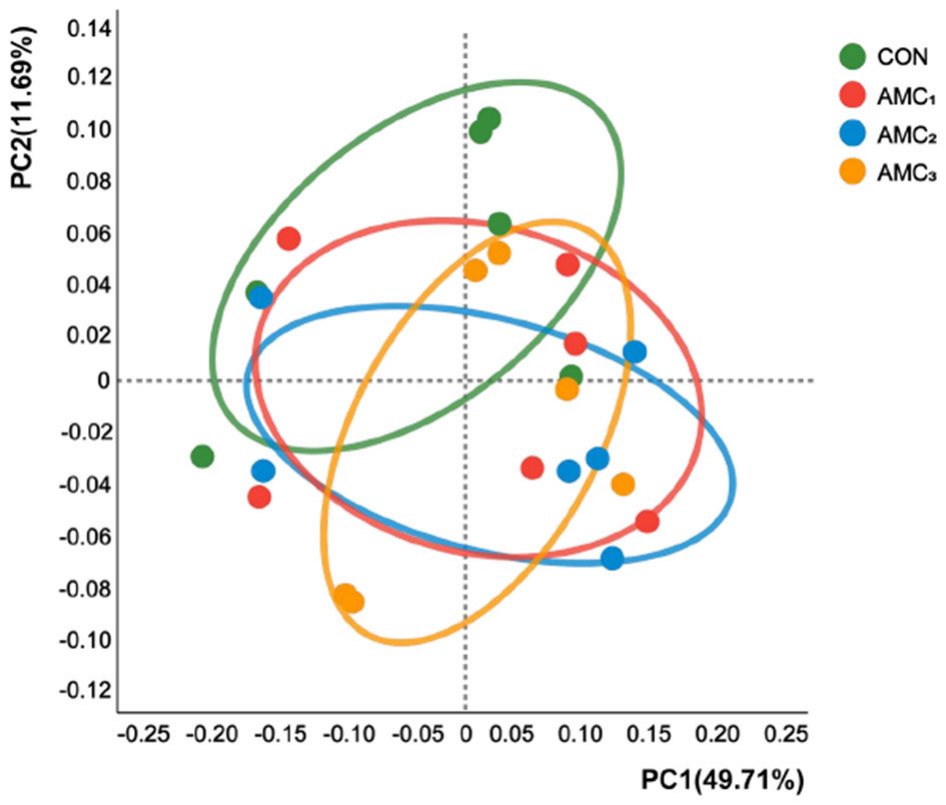
Principal coordinate analysis (PCoA) combined with permutational multivariate analysis of variance (PREANOVA) was calculated based on the ASV level and Bray–Curtis distances. CON, no supplementation; AMC_1_, the AMC concentration in the substrate is 1 mL/kg; AMC_2_, the AMC concentration in the substrate is 2 mL/kg; AMC_3_, the AMC concentration in the substrate is 4 mL/kg.

In addition, there was no distinct separation between the different supplementation groups and the CON group in the PCoA plot based on Bray–Curtis staining (Figure 3, *p* > 0.05). These results indicated that there were no significant differences in the species diversity of rumen fluid bacteria among the different concentrations of AMC.

**Figure 4:**
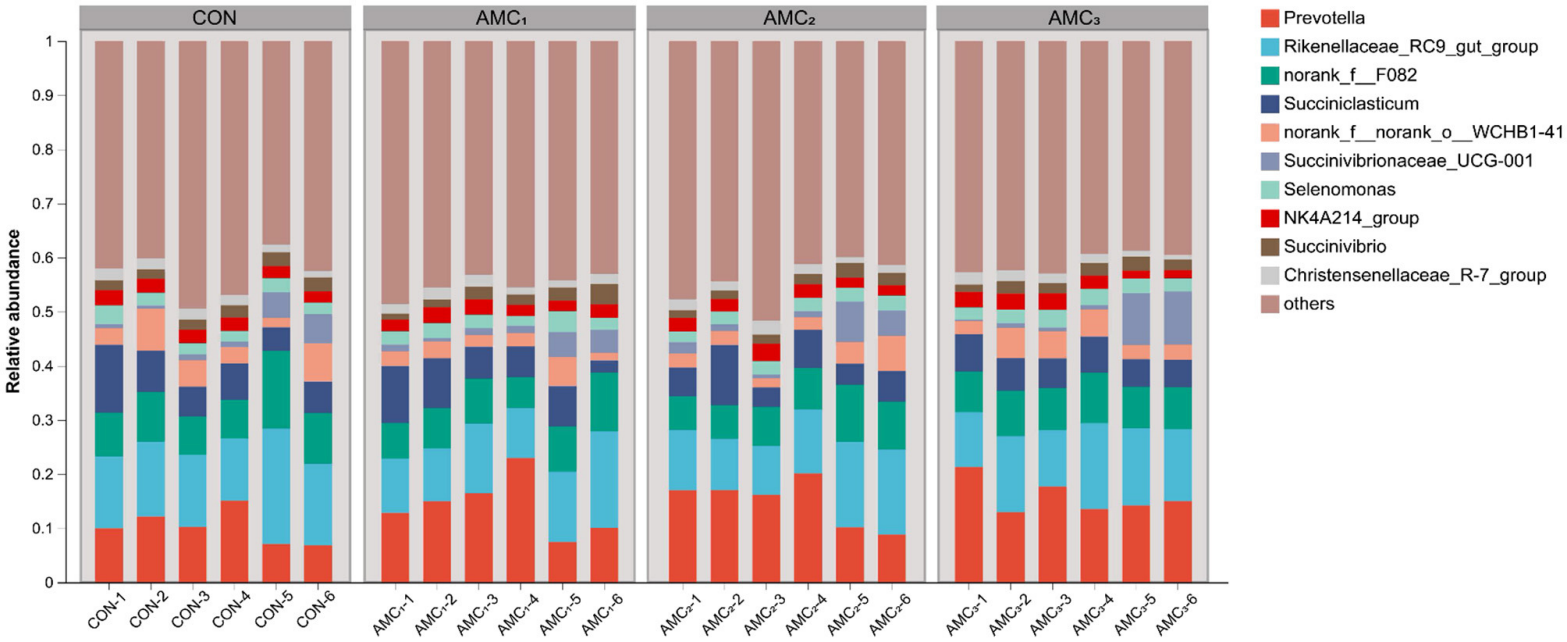
Genera composition of the microbiota from *in vitro* fermentation technique; CON, no supplementation; AMC_1_, the AMC concentration in the substrate is 1 mL/kg; AMC_2_, the AMC concentration in the substrate is 2 mL/kg; AMC_3_, the AMC concentration in the substrate is 4 mL/kg.

Figure 4 shows an overview of the genus composition of the microbiota. The abundances of *Prevotella, Rikenellaceae-RC9-gut-group*, and norank-f–F082 were found to be enriched in different groups.

**Figure 5:**
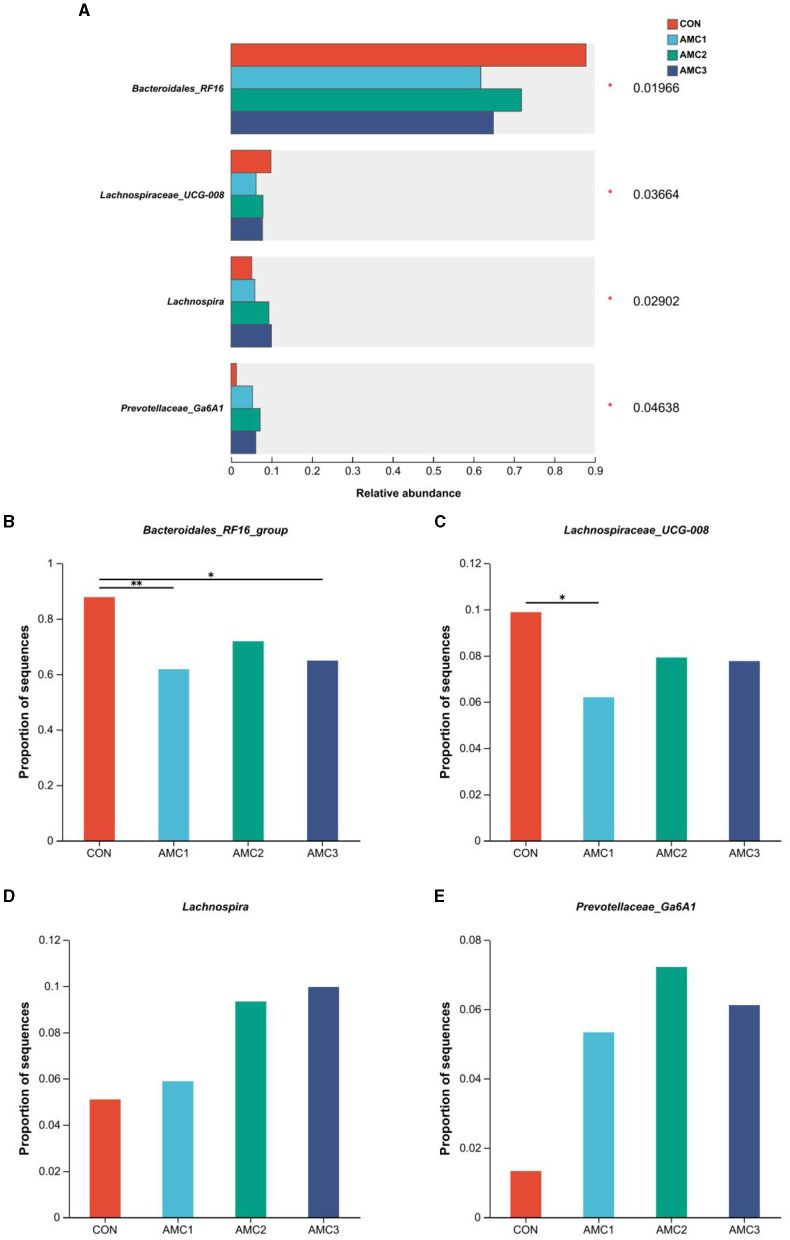
Bacterium with significant differences in species abundance at the genus level **(A)**, relative abundance and *p*-value cut-off were < 0.05; Analysis of differences in bacteria between any two groups **(B–E)**: *0.01 < *p* ≤ 0.05, **0.001 < *p* ≤ 0.01, ***p ≤ 0.001; CON, no supplementation; AMC_1_, the AMC concentration in the substrate is 1 mL/kg; AMC_2_, the AMC concentration in the substrate is 2 mL/kg; AMC_3_, the AMC concentration in the substrate is 4 mL/kg.

Figure 5 shows the microbial composition at the genus level under the different AMC treatments. As shown in Figure 5B, the relative abundance of Bacteroidales-RF16 in the AMC_1_ and AMC_3_ groups was significantly lower than that in the CON group (*p* < 0.05). Furthermore, the relative abundance of Lachnospiraceae UCG-008 in the AMC_1_ group was significantly lower than that in the CON group (Figure 5C, *p* < 0.05). Regarding Prevotellaceae-Ga6A1 and Lachnospira, different treatments revealed a significant impact on their abundance (Figure 5A, *p* < 0.05). However, the difference between the groups was not statistically significant (Figure 5D, *p* > 0.05). The relative abundance of the CON group was lower than that of the other treatment groups.

## 4 Discussion

In this study, we focused on one of the prerequisites for normal rumen fermentation, which is the normal pH range of rumen fluid (5.5–7.5) (24). By adding AMC at different concentrations during *in vitro* fermentation of the rumen fluid, we observed that the pH of the rumen fluid, when fermented *in vitro* with different concentrations of AMC, ranged from 5.8 to 7.0, which is consistent with the optimal range of rumen pH. Notably, the addition of 1–2 mL/kg of AMC significantly elevated rumen pH at 6 and 24 h, suggesting that AMC had a significant buffering effect on rumen pH, particularly within the first 24 h of supplementation. Furthermore, the buffering effect of AMC stabilized after 24 h, as both the control and treatment groups exhibited a tendency toward stable rumen pH levels after this time point.

GP_48_ and HT are important indicators of rumen fermentation capacity and nutrient digestibility. Through our experiments, we found that AMC had a significant impact on the HT, consistent with the results of the pH. Specifically, when the rumen microbiota metabolizes to produce excess hydrogen ions, AMC can neutralize these ions, maintaining the solution's pH at a relatively stable level. This provides a suitable environment for promoting microbial activity and gas production processes.

VFAs, the main products of rumen fermentation, serve as the main energy sources and raw materials for synthetic and milk fats. Acetic and butyric acids are mainly used for milk fat synthesis, whereas propionic acid serves as a precursor for glucose synthesis and can competitively consume hydrogen to reduce methane production (14). Propionic acid is rapidly oxidized in the liver to produce energy. Similar to other short-chain fatty acids, propionic acid is a product of intestinal microbial fermentation of fiber and other indigestible carbohydrates, which is crucial for maintaining intestinal health and function. Valeric acid, isovaleric acid, and isobutyric acid, collectively categorized as short-chain VFAs with four to five carbon atoms, are referred to as branched-chain VFAs (25). The results in the present study indicated that all VFAs in the fermentation broth, except isobutyric acid, showed a quadratic change with the increasing concentration of AMC. As the AMC concentration increased, the acetic, propionic, butyric, valeric, and isovaleric acid contents increased and then decreased, and the A/P ratio first decreased and then increased. Although the ammonia nitrogen concentration remained unchanged, the total VFA concentration increased in the group supplemented with bicarbonate, indicating that the addition of a combination of buffer altered the liquid turnover and the rumen fermentation mode (26), which was beneficial for providing energy for ruminants. This trend can be attributed to an increase in *Prevotellacea-Ga6A1*. Previous studies have shown that Prevotella metabolizes hemicellulose, pectin, and proteins, with acetic and formic acids being the main fermentation products (27). The increase in these short-chain fatty acids led to a trend toward propionic acid-type fermentation in the rumen, indicating that more propionic acid provides energy through gluconeogenesis, which is particularly important for maintaining the energy balance in ruminants. High levels of propionic acid can inhibit milk fat synthesis, but this needs to be validated via *in vitro* experiments. These findings support the significant role of buffering agents in maintaining energy balance and promoting rumen health in ruminants.

The competitive dynamics observed between fiber-degrading bacteria in the phylum Bacillota and the genus *Prevotella*, with an increased relative abundance of *Prevotellacea-Ga6A1*, correspond to our results and suggest an inhibitory effect on the growth of *Lachnospiraceae UCG-008*. *Lachnospira* can also degrade polysaccharides and fiber contents to produce acetic acid. The observed discrepancies in ADF and VFA may be attributed to an increase in *Lachnospira* abundance (28). This indicated that AMC supplementation promoted the growth of beneficial bacteria in rumen microorganisms and the reproduction and metabolism of acetic acid-producing bacteria. AMC facilitated the fermentation and decomposition of carbohydrates, thereby promoting the metabolism and absorption of nutrients by dairy cattle.

NH_3_-N is produced by the fermentation of protein, non-protein nitrogen, and other nitrogenous compounds, and this can reflect the degree of rumen nitrogen metabolism (29). Inappropriate concentrations of NH_3_-N affect animal health. The appropriate range of rumen NH_3_-N concentration had been reported to be 6–30 mg/dL (30), and the result of the present study showed that the concentration of NH_3_-N are all within a reasonable range. MCP is the predominant nitrogen source for dairy cattle, contributing 60–80% of the required protein. This reflects the microbial utilization of NH_3_-N and indicates the abundance of microorganisms (31). Ample nitrogen sources, provision of VFAs as carbon scaffolds, and fermentation of organic matter have a collaborative effect on the synthesis efficiency and quantity of MCP (32). In this study, the addition of AMC significantly influenced the concentration of NH_3_-N. Additionally, NH_3_-N and MCP exhibited a quadratic trend with increasing AMC, indicating that AMC promoted rumen microorganisms to comprehensively utilize nutrients in the fermentation substrate.

The PCoA analysis revealed no significant differences in the microbial community structure between the treatment and control groups, suggesting the relatively mildness of the buffer. It is essential to emphasize that the subtle effects of the buffering agents do not imply a lack of impact on the microorganisms in all scenarios. These variations may be associated with the differences between individual samples, sample sizes, and the simulated environment of the *in vitro* experiments. It is imperative to conduct further *in vivo* experiments to validate the efficacy of this buffer.

## Data availability statement

The raw data supporting the conclusions of this article will be made available by the authors, without undue reservation.

## Ethics statement

The animal study was approved by the Institutional Animal Care and Use Committee of China Agricultural University. The study was conducted in accordance with the local legislation and institutional requirements.

## Author contributions

SL: Methodology, Software, Visualization, Writing – original draft, Writing – review & editing. BX: Data curation, Formal Analysis, Methodology, Writing – review & editing. HJ: Formal Analysis, Methodology, Visualization, Writing – review & editing. SL: Funding acquisition, Writing – review & editing.
